# Guyton’s hemodynamic mosaic: crafting fluid management with precision

**DOI:** 10.1186/s13613-024-01416-w

**Published:** 2025-01-03

**Authors:** Rogerio Da Hora Passos, Luciano Ribeiro Pereira Silva, Leonardo Van De Wiel Barros Urbano Andari, Uri Adrian Prync Flato, Murillo Santucci Cesar Assunção, Thiago Domingos Corrêa

**Affiliations:** Intensive Care Department, Hospital Israelita Albert Einstein, Av. Albert Einstein, 627/701 – Morumbi, São Paulo, CEP: 05651-901 SP Brasil

## Abstract

Sheldon Magder’s article on applying Arthur Guyton’s principles to clinical fluid management provides valuable insights into optimizing hemodynamics in critically ill patients. While emphasizing the role of right atrial pressure (RAP) in assessing cardiac output, challenges arise due to RAP’s variable accuracy and the oversimplification of cardiovascular dynamics. Integrating RAP with dynamic assessments and bedside ultrasound can enhance fluid management strategies. Future research should aim to improve RAP’s predictive accuracy and validate its clinical utility for individualized patient care.

Dear Editor,

I read with great interest the article by Sheldon Magder on the application of Arthur Guyton’s principles to control cardiac output for clinical fluid management [1]. Magder provides a comprehensive review of how understanding venous return and cardiac function through right atrial pressure (RAP) can guide clinical decisions regarding fluid therapy in critically ill patients. His emphasis on the interaction between venous return and cardiac function using Guyton’s framework offers valuable insights into optimizing hemodynamic management.

Magder adeptly highlights the pivotal role of RAP in assessing the adequacy of cardiac output and the potential risks associated with fluid administration, particularly in patients with compromised cardiac function or altered venous return. His explanation of venous collapse pressures and the implications for fluid responsiveness is particularly insightful for clinicians managing fluid resuscitation strategies.

However, the application of Guyton’s principles in clinical practice warrants careful consideration of several challenges. One key challenge lies in the assumption that RAP accurately reflects central venous pressure and thus venous return. Variations in vascular compliance, intra-abdominal pressures, and the influence of positive pressure ventilation can significantly alter the relationship between RAP and true venous return, complicating the interpretation of hemodynamic status [2, 3].

Furthermore, Guyton’s model simplifies the complex interplay between cardiac output and venous return into linear relationships, potentially oversimplifying the dynamic nature of cardiovascular physiology. Individual patient variability in vascular tone, myocardial compliance, and response to fluid therapy may not always conform to the predicted outcomes based solely on RAP measurements [2]. 

From a practical standpoint, the clinical utility of RAP as a singular marker for fluid responsiveness may be limited by its static measurement in a dynamic clinical environment. Real-time adjustments in fluid therapy require consideration of additional hemodynamic parameters such as stroke volume variation, pulse pressure variation, and dynamic indices of fluid responsiveness, which complement rather than replace RAP measurements [4, 5].

To enhance the clinical application of RAP, integrating it with dynamic assessments like stroke volume variation and response to fluid challenges can provide a more comprehensive hemodynamic picture. Additionally, employing bedside ultrasound for real-time visualization of venous congestion and assessing collapsibility indices can augment the accuracy of RAP-guided fluid management strategies [5]. 

In conclusion, while Guyton’s principles provide a foundational framework for understanding cardiovascular physiology, their application in clinical practice necessitates a nuanced approach. Magder’s review underscores the importance of integrating theoretical concepts with practical clinical judgment and the use of multimodal hemodynamic monitoring. Future research should explore ways to enhance the predictive accuracy of RAP-based assessments and validate its utility in guiding individualized fluid management strategies.

## Data Availability

Not applicable.

## Abbreviations

RAP: Right atrial pressure

## References

[1] Magder S. The use of Guyton’s approach to the control of cardiac output for clinical fluid management. Ann Intensive Care. 2024;14:105. 10.1186/s13613-024-01316-z. 38963533 10.1186/s13613-024-01316-z PMC11224168

[2] De Backer D, Vincent JL. Should we measure the central venous pressure to guide fluid management? Ten answers to 10 questions. Crit Care. 2018;22(1):43. 10.1186/s13054-018-1959-3. PMID: 29471884; PMCID: PMC5824587. 29471884 10.1186/s13054-018-1959-3 PMC5824587

[3] Berger D, Takala J. Determinants of systemic venous return and the impact of positive pressure ventilation. Ann Transl Med. 2018;6(18):350. 10.21037/atm.2018.05.27. PMID: 30370277; PMCID: PMC6186556. 30370277 10.21037/atm.2018.05.27 PMC6186556

[4] Horejsek J, Kunstyr J, Michalek P, Porizka M. Novel methods for Predicting Fluid responsiveness in critically Ill Patients-A Narrative Review. Diagnostics (Basel). 2022;12(2):513. 10.3390/diagnostics12020513. PMID: 35204603; PMCID: PMC8871108. 35204603 10.3390/diagnostics12020513 PMC8871108

[5] Monnet X, Shi R, Teboul JL. Prediction of fluid responsiveness. What’s new? Ann Intensive Care. 2022;12:46. 10.1186/s13613-022-01022-8. 35633423 10.1186/s13613-022-01022-8 PMC9148319

